# Preferences for coordinated care for rare diseases: discrete choice experiment

**DOI:** 10.1186/s13023-024-03353-0

**Published:** 2024-09-09

**Authors:** Stephen Morris, Holly Walton, Amy Simpson, Kerry Leeson-Beevers, Lara Bloom, Amy Hunter, Angus I. G. Ramsay, Naomi J. Fulop, Lyn S. Chitty, Joe Kai, Alastair G. Sutcliffe, Maria Kokocinska, Larissa Kerecuk, Christine A. Taylor, Pei Li Ng

**Affiliations:** 1 Primary Care Unit, Department of Public Health & Primary Care, University of Cambridge, East Forvie Building, Forvie Site, Robinson Way, Cambridge, CB2 0SR UK; 2 Department of Applied Health Research, University College London, England, UK; 3 Oxford Brookes University, Oxford, UK; 4 Alström Syndrome, England, UK; 5 The Ehlers-Danlos Society, London, UK; 6 Genetic Alliance UK, London, UK; 7 London North Genomic Laboratory Hub, Great Ormond Street NHS Foundation Trust, London, UK; 8 UCL Great Ormond Street Institute of Child Health, London, UK; 9 Division of Primary Care, University of Nottingham, Nottingham, UK; 10 Birmingham Women’s and Children’s NHS Foundation Trust, Birmingham, UK; 11 NIHR Clinical Research Network West Midlands, Birmingham, UK

**Keywords:** Rare diseases, Rare conditions, Care coordination, Discrete choice experiment, Preferences

## Abstract

**Background:**

Evidence suggests that coordination of care for people affected by rare diseases is poor. In order to improve the way that care is coordinated it is necessary to understand the preferences of people affected by these conditions, and providers. The aim of this study was to examine patient, parent and carer, and health care professional preferences for different attributes of care coordination for people affected by rare diseases. We conducted a discrete choice experiment using online surveys. There were no restrictions on participants in terms of rare conditions, demographic factors other than age, or geographical location within the UK. Choice scenarios were based on the following attributes: annual cost of attending appointments; access to health records; access to clinical expertise; support of a care coordinator; access to a specialist centre; and, the existence of a documented plan for emergency care. Data were analysed using alternative-specific conditional logit regression models.

**Results:**

Valid responses were obtained from 996 individuals (528 patients, 280 carers, 188 health care professionals) between August and December 2019. All attributes significantly influenced the type of service respondents preferred. Patients, carers and health professionals’ preferences for care coordination were influenced by: the cost of attending appointments; access to health records; clinical expertise; role of care coordinators; access to specialist centres; and the existence of plan for emergency care. There were no statistically significant differences in the preferences between patients and carers. Preferences of health professionals differed to those of patients and carers. Both patients and carers selected responses which granted them a greater degree of autonomy in relation to the role of care coordinators, whereas health professionals preferred services where care coordinators had more autonomy. Health care professionals also expressed a stronger preference for a documented formal emergency plan to be in place.

**Conclusions:**

The findings highlight that people value better coordinated care, in line with policy documents emphasising commitments to coordinated care for people affected by rare diseases. This study highlights the factors that could be included in service provision as ways of improving the coordination of care for people affected by rare diseases.

**Supplementary Information:**

The online version contains supplementary material available at 10.1186/s13023-024-03353-0.

## Background

Walton et al. defined coordinated care in the context of rare conditions as follows:

“*Coordination of care involves working together across multiple components and processes of care to enable everyone involved in a patient’s care (including a team of healthcare professionals*,* the patient and/or carer and their family) to avoid duplication and achieve shared outcomes*,* throughout a person’s whole life*,* across all parts of the health and care system*,* including: care from different healthcare services[…]*,* care from different healthcare settings[…*,*] care across multiple conditions or single conditions that affect multiple parts of the body*,* the movement from one service*,* or setting to another. Coordination of care should be family-centred*,* holistic (including a patient’s medical*,* psychosocial*,* educational and vocational needs)*,* evidence-based*,* with equal access to coordinated care irrespective of diagnosis*,* patient circumstances and geographical location*” [1].

There is evidence to suggest that poor coordination of care is a problem faced by many people affected by rare conditions. For example, information on test and procedure results and treatment may not be shared effectively between services, patients and families frequently have to attend multiple clinics and travel significant distances to them, many patients do not have access to a care coordinator or advisor, and there is often limited access to specialist centres [2]. Such data illustrate the heavy burden poor care coordination places on patients and families dealing with rare diseases.

The lack of coordinated care can have major impacts on patients and families affected by rare diseases. Simpson et al. [3]. found that uncoordinated care had an impact on physical health (including fatigue), financial impact (including loss of earnings and travel costs), and psychosocial impact (including disruption to school, work and emotional burden).

In terms of the policy background and context to this study, the importance of better coordination of care for people affected by rare conditions has been highlighted by the UK governments. In 2013 the Department of Health, Northern Ireland Executive, Scottish Government and the National Assembly for Wales published The UK Strategy for Rare Diseases [4], which said it was essential to coordinate care for people with rare diseases. It also stated that more needed to be done to improve coordination, and that research was needed on how care for people with rare diseases should be coordinated. In the progress report from the All Party Parliamentary Group on Rare, Genetic and Undiagnosed Conditions (February 2017) it was noted: “Patient care continues to be poorly co-ordinated” [5]. The UK government, highlighted the problem of coordinated care for people affected by rare conditions more recently in the UK Rare Diseases Framework, published in 2021 [6]. This stated that coordination of care was one of the top challenges facing people affected by rare diseases, and better coordination was listed as one of the four top priorities to be addressed over the next five years. It was also listed as one of the four major challenges facing the rare disease community. In a ‘National Conversation’ survey of 6293 members of the UK rare diseases community conducted in 2019, coordination of care was identified as the top challenge by 16% of patients, 19% of families and carers, 11% of rare disease patient organisations, and by 18% of health care professionals [6]. Recently, there have been a range of policy drivers introduced throughout the UK that outline actions plans for, among other things, how the co-ordination of care for people affected by rare diseases ought to be improved [7–10]. Within the European Union (EU), there is also significant policy interest and activity in the area of care co-ordination [11]. For example, there has been a focus on strengthening cooperation and coordination to improve access to knowledge, diagnosis and treatment of rare diseases via the 24 European Reference Networks [12], which create a clear governance structure for knowledge sharing and care coordination across the EU [13].

In order to improve the way that care is coordinated it is necessary to understand the preferences of people affected by rare diseases and providers – how they would like care for rare conditions to be coordinated. The aim of this study was to examine patient, parent and carer, and health care professional preferences for different attributes of care coordination for people affected by rare diseases, and how these preferences varied between groups. To our knowledge there are no previous studies that have examined this topic in the context of rare conditions, though similar work has been conducted in the care of older people [14].

## Methods

### Overview of approach

Preferences were explored using a discrete choice experiment (DCE) [15]. In DCEs, respondents are typically presented with a series of questions, asking them to choose between two or more alternatives that describe a service in terms of a set of characteristics or attributes. This allows the attributes of a service that respondents prefer to be evaluated, as well as the trade-offs they are willing to make between these attributes. DCE good-practice guidelines were followed for the design of this study and the analysis [16].

#### Survey sampling

Three groups of participants were eligible to complete the DCE: patients aged ≥ 18 years affected by a rare condition; parents and carers aged ≥ 18 years of children or adults with rare conditions; and, health care professionals (doctors, nurses and allied health professionals) involved in the care of people with rare conditions. We aimed to recruit 300 participants for each group. Although no consensus exists regarding sample size calculations for DCEs because of their complexity [17], this sample size is similar to previous studies [18].

There were no restrictions on participants in terms of the rare condition they were affected by, demographic factors (other than age ≥ 18 years), or geographical location within the UK. We deliberately did not sample from specific rare conditions, nor limit the range of rare conditions we included, in order to identify as many different models of coordination as possible, and include as broad a range of experiences and preferences with regards to care coordination as possible. A complete sample frame of all adults living with a rare condition in the UK does not exist: the total number of people living with a rare condition, their contact details and their socio-demographic characteristics such as age, gender, highest education level and location of residence are unknown. For these reasons, purposive snowball sampling was used. Routes to accessing patients and parents/carers were determined with the study’s Patient and Public Involvement Advisory Group (PPIAG).

Participants were accessed via patient and provider networks and organisations, including Rare Disease UK (which has more than 2000 registered supporters including academics, clinicians, industry, individual members and patient organisations [19]); Genetic Alliance UK (a national alliance of organisations with a membership of more than 180 charities supporting patients and families affected by genetic disorders [20]); and SWAN UK (Syndromes Without A Name; a support network for families of children and young adults with undiagnosed genetic conditions in the UK run by Genetic Alliance UK [21]). We also recruited patients and parents/carers via six major care providers, where research coordinators at each site identified potential participants, asked if they were willing to participate, and provided further details on how to do this if so, as above. Health care professionals were recruited using the same routes described above for patients and parents/carers. In addition, we contacted the British Society of Genetic Medicine and its constituent organisations and Special Interest Groups [22], and the NIHR Clinical Research Network: Genetics (now Genomics and Rare Diseases) [23]. These organisations circulated details of the survey to their members via their mailing lists.

An independent survey company created an electronic version of the survey using a bespoke online platform. Potential participants were sent a weblink to the survey either by email or social media that had an embedded Participant Information Sheet at the start. From this, participants were asked to click to another webpage to access the survey, and were informed that by doing so they consented to take part in the study. They were also told that they did not have to take part if they did not want to. Participants had a 48-hour window where they were able to suspend completion of the questionnaire, if they wished to do so, and then to resume where they left off at a time that was convenient to them. The correspondence containing the weblink also included an offer to send hard copies of the questionnaire by post or email or to complete it verbally over the telephone with a researcher.

### Attributes and levels

The attributes and levels used in the DCE describing elements of coordinated care were identified using three sources. First, a scoping review of 154 reviews of coordinated care for rare conditions [1]. This identified components of coordinated care within the context of rare diseases. Second, we ran three focus groups involving patients over 18 years affected by a rare condition, parents/carers of children and adults affected by a rare condition, and health care professionals involved in the treatment of rare conditions. One focus group was conducted virtually involving four patients and three carers; two were conducted face-to-face, one involving four health care professionals, the other involving two patients and four parents/carers. Third, we ran 15 one-to-one telephone or Skype interviews involving seven patients and eight parents/carers. In the focus groups and interviews we asked respondents to identify the characteristics of coordinated care that mattered most to them. Analyses of these data identified six attributes reflecting the extent of care coordination for rare conditions: cost to patients and carers of attending all appointments over one year; access to health records; clinical expertise; role of the care coordinator; access to a specialist centre; and, having a documented emergency care plan (Table 1), and these were selected as the final attributes to be included in the DCE. Credible levels for each attribute were chosen based on either known characteristics (e.g., the presence of that aspect of care coordination), or feedback from the interviews and focus groups (e.g., preferred interaction with care coordinators, costs for attending appointments). Descriptions were developed for each attribute to help participants understand the nature of each attribute that they were being asked to consider. All material was scrutinised by the study PPIAG, who agreed the attributes and levels and made changes to the descriptions.

**Table 1:** Attributes and levels used in the discrete choice experiment

Attribute	Description	Levels					
Cost to patients and carers of attending all appointments over one year	Describes the cost to patients and carers of attending all health care appointments over one year (including travel costs, time off work, childcare costs, subsistence)	£200	£400		£1000		£2000
Access to health records	Describes the way in which health records are shared by different health professionals in the same centre or across different health settings	Health records are not shared; test results and clinic letters are sent through the post			Electronic health records are immediately accessible to staff		
Clinical expertise	The type of medical professional who is the lead consultant and makes the majority of decisions regarding medical care	The lead consultant is a medical expert in your specific condition			The lead consultant is a medical expert in the area of the body primarily affected by your condition (e.g., neurologist)		
Role of care coordinator	Describes the amount of involvement of a formal care coordinator who is a health care professional	Care is provided without the support of a care coordinator		Care is entirely coordinated on your behalf by a care coordinator		You have a named care coordinator and you decide how they support you	
Access to Specialist Centre	A Specialist Centre enables patients to see a number of health professionals in one visit. Generally, they will be experts in rare and undiagnosed conditions. Non-health professionals may also see patients at the same centre	You do not have access to a specialist centre			A specialist centre is available		
Documented emergency Plan	A formal emergency plan describes the correct treatment which should be provided in urgent situations and contact details for a health professional who has knowledge of the specific condition.	There is a documented emergency plan in place			No documented emergency plan exists		

### Questionnaire design

**Fig. 1:**
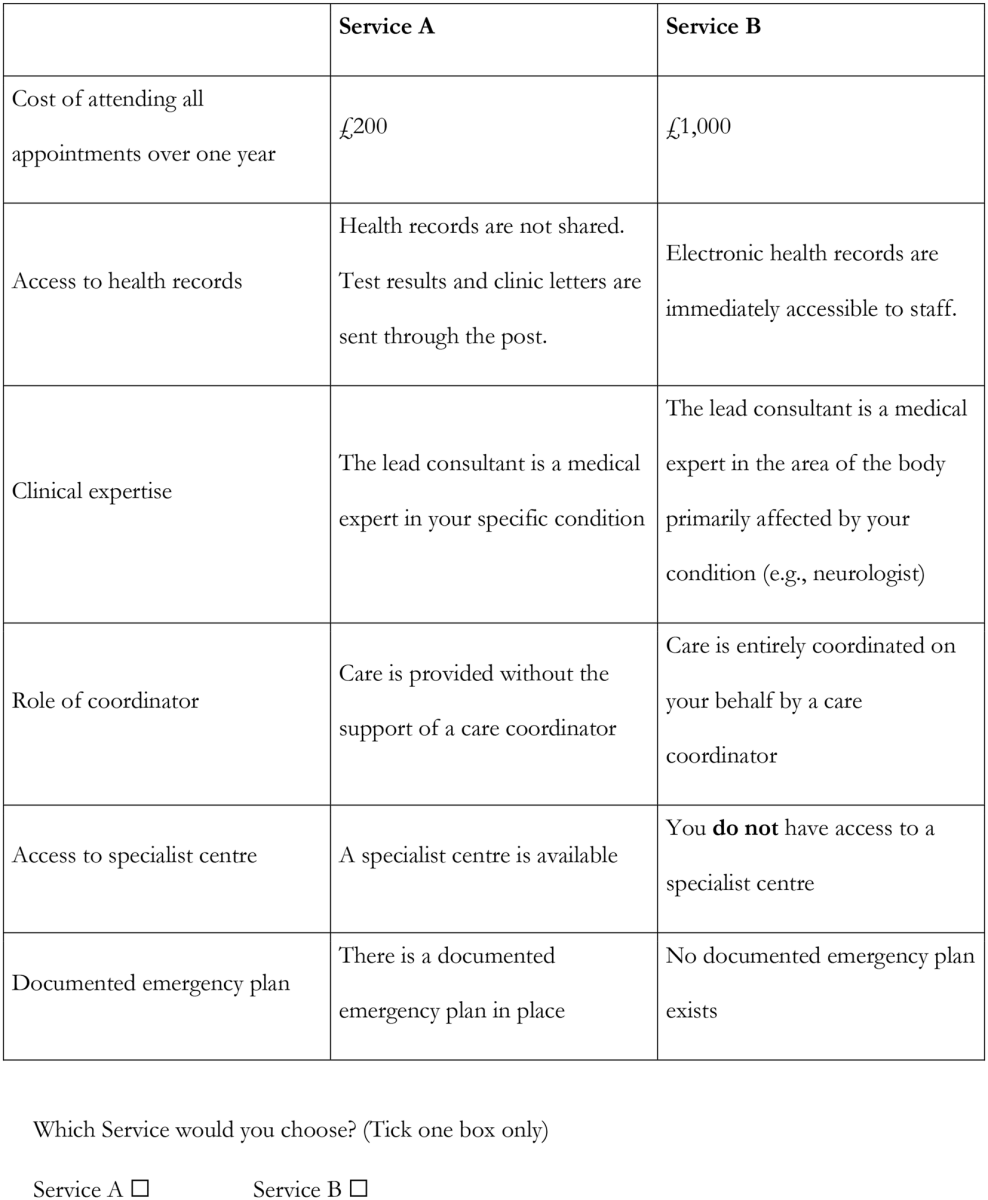
Example of DCE question

Respondents were asked to choose their preferred option from a series of pairwise choices, asking in which of two fictitious services they would prefer to receive their care (in the case of patients), or the person they care for (parents/carers), or their patients (health care professionals). Each service was described by a combination of different levels of the attributes; Fig. 1 shows an example of a DCE question. An opt-out or ‘neither’ option was not included as people are unlikely to choose none of the available options given current levels of service provision. The number of potential combinations of attributes with four two-level attributes, one three-level attributes and one four-level attribute is 192 (2^4^ × 3^1^ × 4^1^). With two options to choose from in each choice question, this gives a possible 36 672 choices (192 × 191). To reduce the number of choices to a manageable number, a fractional design was applied using the –dcreate– command in Stata [24], which creates efficient designs for DCEs. The choice set was reduced to 18 scenarios, which were split into three blocks of six, and a third of the respondents in each group were assigned to each block. Hence, nine versions of the DCE questionnaire were used: three for patients, three for parents/carers, three for health professionals. The questionnaire also included a question asking respondents to rank the six attributes according to their overall importance, from 1 (most important) to 6 (least important). Information on demographic, socioeconomic and rare-disease-related experience was also collected. The questionnaire was piloted in 11 respondents (3 patients, 4 carers, 4 health care professionals) in 3 think-aloud interviews (2 carers, 1 health care professional) and 8 providing written feedback. This resulted in minor improvements being made to the wording of the questionnaire.

### Data analysis

Descriptive statistics for the characteristics of respondents in each group were computed. Responses to the ranking questions were analysed graphically. The DCE data were analysed using alternative-specific conditional logit regression models in which the outcome was service preference (A or B) and the variables in the equation were the individual attributes. A constant term was not included. Models were run for each group separately and differences in preferences between the groups were tested by comparing the coefficients for each group using χ^2^ tests. Where the coefficients were not jointly different between groups, those groups were combined in subsequent analyses. The relative importance of each attribute was calculated as the difference in the coefficients between the best or most preferred level of each attribute and the worst or least preferred level of the same attribute [25]. We calculated marginal rates of substitution (MRS) with respect to the cost attribute (cost to patients and carers of attending all appointments during 1 year); this allows direct assessment of how much of one attribute participants are willing to trade for one unit of another attribute, and therefore enables a comparison of different attributes on a common scale. Using the cost attribute as the denominator means that participants’ preferences and the trade-offs can be evaluated in terms of willingness to pay. The standard error of the MRS was calculated using the delta method. The results of the regression analysis were used to calculate the predicted probabilities of choosing coordinated services compared with no coordination. No coordination was defined as: cost to patients and carers of attending all appointments during 1 year were £1000; health records were not shared; the lead consultant was a medical expert in the area of the body primarily affected by the patient’s condition (e.g. neurologist); care was provided without the support of a care coordinator; a specialist centre was not available; there was not a documented emergency plan in place. In each coordination scenario, costs remained fixed at £1000 (coordination has no impact on costs) and the following potential characteristics of a coordinated service were amended individually and then jointly: electronic health records were immediately accessible to staff; the lead consultant was a medical expert in the patient’s specific condition; the patient/carer decided how they wished to be supported by the care coordinator; a specialist centre was available; there was a documented emergency plan in place. We recalculated the predicted probabilities first assuming no coordination was associated with high costs (£2000) and coordination was associated with low cost (£200), and second assuming no coordination was associated with low costs and coordination was associated with high cost. All analyses were undertaken using the software package Stata^®^ version 15.0 (StataCorp, College Station, Texas, USA).

## Results

### Responses and sample

In total 996 responses to the DCE section of the survey were received, 528 from patients, 280 from carers and 188 from health care professionals, between August to December 2019. It was not possible to estimate a response rate for each group because the survey was sent via multiple overlapping distribution routes. Of 528 (adult) patients with a rare condition the modal age band was 45 to 54 years and over 80% were female (Table 2). Over 95% were diagnosed with a rare disease (as opposed to being undiagnosed), and diagnoses had been confirmed by a genetic test in around 30% of respondents. Numerous rare conditions were reported, the most prevalent being sarcoidosis and Behcet’s Syndrome. Most patients reported they lived with a spouse or partner (55%). The modal age category of carers was 35 to 44 years (34%), and in around two-thirds of cases the patient being cared for was under 18 years. Around 80% of carers who responded were female. Most patients of the carers had been diagnosed with a rare disease (8% were undiagnosed) and in 60% of cases the diagnosis had been confirmed with a genetic test. The most common rare conditions in the sample were Tracheo-Oesophageal Fistula and Behcet’s Syndrome. In two-thirds of cases the carer was the parent of the patient affected by the rare condition and in the vast majority of cases the carer lived with the patient. Among both patients and carers around 90% of both groups were from the white ethnic group, and from a range of educational backgrounds, with having a degree or higher degree being the modal education category. Across the 188 health care professionals, just over half reported having specific clinical expertise in rare diseases, and they worked across a range of areas with patients with rare conditions. Around 40% were hospital doctors and 20% were nurses or midwives. All three groups were spread across regions of the UK.

**Table 2:** Descriptive characteristics by group

	Patients (*N* = 528)	Parents/carers (*N* = 280)	HCPs (*N* = 188)
	*n* (%)	*n* (%)	*n* (%)
**Age of patient (years)**			
0 to 5	-	66 (23.6)	-
6 to 12	-	81 (28.9)	-
13 to 17	-	34 (12.1)	-
18 to 24	21 (4.0)	33 (11.8)	-
25 to 34	75 (14.2)	18 (6.4)	-
35 to 44	94 (17.8)	8 (2.9)	-
45 to 54	124 (23.5)	11 (3.9)	-
55 to 64	115 (21.8)	12 (4.3)	-
65 to 74	66 (12.5)	4 (1.4)	-
75+	14 (2.6)	1 (0.4)	-
Missing	19 (3.6)	12 (4.3)	-
**Age of parent/carer (years)**			
18 to 24	-	5 (1.8)	-
25 to 34	-	36 (12.9)	-
35 to 44	-	94 (33.6)	-
45 to 54	-	86 (30.1)	-
55 to 64	-	36 (12.9)	-
65 to 74	-	11 (3.9)	-
75+	-	1 (0.4)	-
Missing	-	11 (3.9)	-
**Sex**			
Female	434 (82.2)	235 (83.9)	-
Male	73 (13.8)	32 (11.4)	-
Other	2 (0.4)	1 (0.4)	-
Missing	19 (3.6)	12 (4.3)	-
**Diagnosed with rare disease**			
Yes	513 (97.2)	257 (91.8)	-
No (undiagnosed)	15 (2.8)	23 (8.2)	-
**Diagnosis confirmed with genetic test**			
Yes	155 (29.4)	167 (59.6)	-
No	258 (53.9)	68 (24.3)	-
Unsure	73 (13.8)	22 (7.9)	
Not applicable (undiagnosed)	15 (2.8)	23 (8.2)	-
**Top 10 most common rare diseases**			
1	Sarcoidosis (67)	Tracheo-Oesophageal Fistula (10)	
2	Behcet’s Syndrome (52)	Behcet’s Syndrome (6)	
3	Idiopathic Intracranial Hypertension (36)	Rett syndrome (5)	
4	Lynch Syndrome (17)	Aplastic Anaemia (4)	
5	Ehlers Danlos Syndrome (12)	Tuberous Sclerosis Complex (4)	
6	IgA Nephropathy (12)	Sarcoidosis (3)	
7	Familial Partial Lipodystrophy (10)	Growth Hormone Deficiency (3)	
8	Ocular Melanoma (8)	Alpha thalassemia X-linked intellectual disability (ATR-X) syndrome (3)	
9	Tarlov Cyst Disease (7)	Idiopathic Intracranial Hypertension (3)	
10	Common Variable Immune Deficiency (6)	Williams Syndrome (3)	
**Parent’s/carer’s relationship to patient**			
Spouse or partner	-	23 (8.21)	-
Parent	-	192 (68.6)	-
Son or daughter	-	41 (14.6)	-
Other	-	24 (8.6)	-
**Parent’s/carer’s living arrangements**			
Lives with patient	-	244 (87.1)	-
Does not live with patient	-	24 (8.6)	-
Missing	-	12 (4.3)	-
**Patient’s living arrangements**			
Lives alone	115 (21.8)	-	-
Lives with a spouse or partner	289 (54.7)	-	-
Lives with family members or friends	99 (18.7)	-	-
Lives with a carer	2 (0.4)	-	-
Missing	23 (4.3)	-	-
**Geographical region**			
East of England	42 (7.9)	17 (6.1)	6 (3.2)
East Midlands	24 (4.5)	17 (6.1)	11 (5.8)
London	52 (9.8)	26 (9.3)	34 (18.1)
North East & Cumbria	23 (4.4)	14 (5.0)	7 (3.7)
Northern Ireland	15 (2.8)	1 (0.4)	1 (0.5)
North West of England	51 (9.7)	34 (12.1)	66 (35.1)
Scotland	60 (11.4)	21 (7.5)	6 (3.2)
South East of England	65 (12.3)	35 (12.5)	9 (4.8)
South West of England	61 (11.5)	26 (9.3)	12 (6.4)
Wales	39 (7.4)	9 (3.2)	1 (0.5)
West Midlands	31 (5.9)	48 (17.1)	25 (13.3)
Yorkshire	35 (6.6)	16 (5.7)	4 (2.1)
Other	8 (1.5)	7 (2.5	4 (2.1)
Missing	22 (4.2)	9 (3.2)	2 (1.1)
**Ethnic group**			
White	473 (89.6)	245 (87.5)	-
Non-white	20 (3.8)	20 (7.2)	-
Missing	35 (6.6)	15 (5.4)	-
**Educational attainment**			
No formal qualifications	18 (3.4)	6 (2.1)	-
O level or GCSE, or equivalent	68 (12.9)	41 (14.6)	-
ONC or BTEC, or equivalent	21 (3.98)	14 (5.0)	-
A level (‘Higher’ in Scotland) or equivalent	35 (6.6)	26 (9.3)	-
Higher education qualification below degree level or equivalent	102 (19.3)	40 (14.3)	-
Degree or higher degree or equivalent	252 (47.7)	130 (46.4)	-
Prefer not to say	32 (6.1)	23 (8.2)	-
**Clinical expertise in rare diseases**			
Yes	-	-	107 (56.9)
No	-	-	81 (43.1)
**Areas of work with patients with rare conditions**			
Diagnosing condition	-	-	117(62.2)
Providing information/signposting, or counselling	-	-	148 (78.7)
Long-term care following diagnosis	-	-	127 (67.5)
Long-term care in the absence of a diagnosis	-	-	109 (58.0)
**Health professional role**			
Allied Health Professional			28 (14.9)
Hospital doctor			78 (41.5)
GP/community doctor			12 (6.4)
Nurse/midwife			39 (20.7)
Clinical academic			24 (12.8)
Other			7 (3.7)

### Simple attribute ranking

**Fig. 2:**
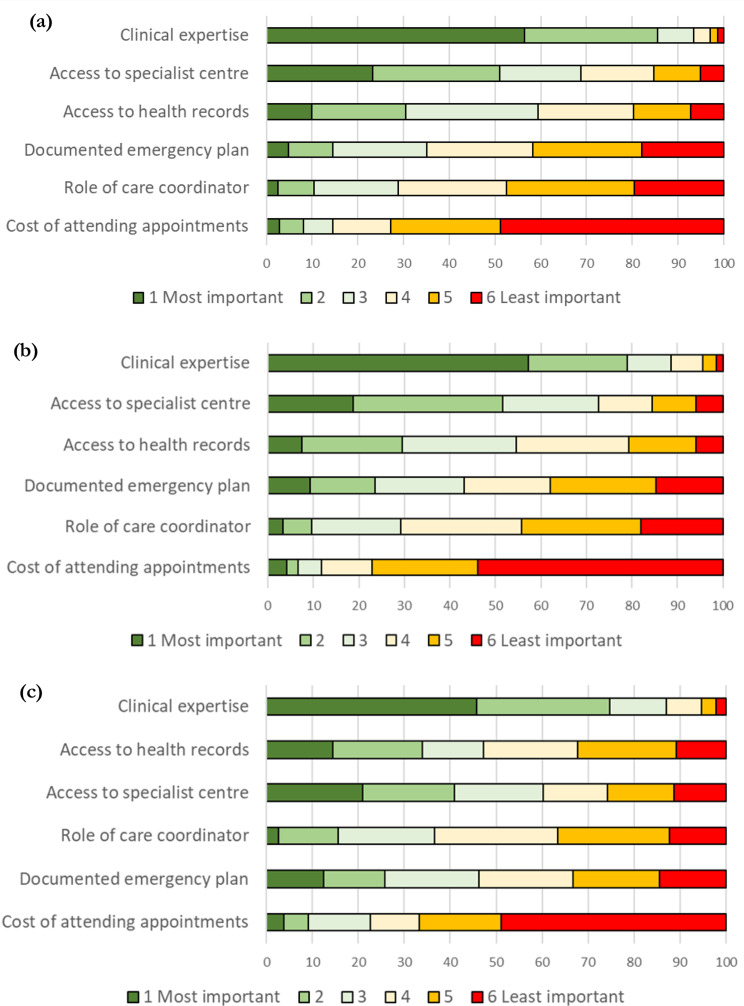
Ranking of attributes by group. (**a**) Patients (512 respondents). (**b**) Parents/Carers (271 respondents). (**c**) Health care professionals (186 respondents)

The responses to the ranking question posed after the DCE questions were examined (Fig. 2); 97% patients and carers and 99% health care professionals provided full responses to this question. Attributes were ranked by likelihood of being selected as the most important factor; using this method of ranking clinical expertise and access to a specialist centre were ranked highly by each group, and the cost of attending appointments was consistently ranked to be the least important factor. There were some differences in how the attributes were ranked for each group.

### Regression analysis

There was no statistically significant difference in the preferences for the attributes between patients and carers (P-value = 0.48) so we reran the analyses for both groups combined (Table 3). Individuals in all groups preferred services with better coordination where: the cost of attending appointments was lower (the coefficients on this variable were negative and statistically significant for all groups); electronic health records were immediately accessible to staff (the coefficients were positive and statistically significant); the lead consultant was a medical expert in the patient’s specific medical condition (the coefficients were positive and statistically significant); care was provided with the support of a care coordinator (the coefficients on both care coordinator categories for this variable were positive and statistically significant); a specialist centre was available (the coefficients were positive and statistically significant); and, there was a documented emergency plan in place (the coefficients were positive and statistically significant). There were some differences between the preferences of patients and parents/carers versus health care professionals (P-value < 0.01). In terms of care coordinators, health care professionals preferred that care was entirely coordinated on behalf of the patient by a care coordinator (the coefficient on the former, 0.461, was larger than the coefficient on the latter, 0.425), whereas patients and carers preferred that they decided how they wished to be supported by the care coordinator (the coefficient on ‘The patient/carer decides how they wish to be supported by the care coordinator’, 0.353, was larger than the coefficient on ‘Care is entirely coordinated on behalf of the patient by a care coordinator’, 0.249) (P-value < 0.01). In terms of emergency plans, all three groups preferred there to be a documented emergency plan in place but the preferences of health care professionals for this was stronger (the coefficient was larger) than for patients and carers (coefficient 0.747 versus 0.369; P-value < 0.01).

**Table 3:** Results of alternative-specific conditional logit regression analysis by group

	Patients (*n* = 528)	Parents/carers (*n* = 280)	Health care professionals (*n* = 188)	*P* ^1^	*P* ^2^	Patients and parents/carers (*n* = 808)
	Coef. (95% CI)	Coef. (95% CI)	Coef. (95% CI) [MRS] {SE}^3^			Coef. (95% CI) [MRS]{SE}^3^
No. of observations	6336	3360	2256			9696
Cost of attending appointments	-0.0003 (-0.0004, -0.0002)	-0.0002 (-0.0003, -0.00004)	-0.0004 (-0.0006, -0.0003)	0.08	0.11	-0.0003 (-0.0003, -0.0002)
Access to health records						
Health records are not shared	-	-	-			-
Electronic health records are immediately accessible to staff	0.630 (0.547, 0.713)	0.728 (0.611, 0.844)	0.761 (0.606, 0.916) [1864]{5634}	0.21	0.17	0.659 (0.592, 0.723) [2442]{7828}
Clinical expertise						
The lead consultant is a medical expert in the area of the body primarily affected by the patient’s condition (e.g., neurologist)	-	-	-			-
The lead consultant is a medical expert in the patient’s specific condition	0.685 (0.571, 0.800)	0.609 (0.437, 0.780)	0.511 (0.309, 0.713) [1252]{4814}	0.33	0.46	0.667 (0.592, 0.727) [2470]{8929}
Role of care coordinator						
Care is provided without the support of a care coordinator	-	-	-			-
Care is entirely coordinated on behalf of the patient by a care coordinator	0.236 (0.080, 0.393)	0.261 (0.043, 0.480)	0.461 (0.196, 0.726) [1131]{5453}	< 0.01	0.15	0.249 (0.122, 0.385) [920]{6576}
The patient/carer decides how they wish to be supported by the care coordinator	0.312 (0.194, 0.430)	0.458 (0.283, 0.634)	0.425 (0.219, 0.632) [1042]{4501}	0.36	0.85	0.353 (0.255, 0.450) [1306]{5739}
Access to specialist centre						
A specialist centre is not available	-	-	-			-
A specialist centre is available	0.676 (0.585, 0.766)	0.699 (0.569, 0.829)	0.735 (0.561, 0.910) [1802]{5660}	0.83	0.77	0.677 (0.604, 0.751) [2509]{8422}
Documented emergency plan						
No documented emergency plan exists	-	-	-			-
There is a documented emergency plan in place	0.359 (0.270, 0.448)	0.393 (0.275, 0.512)	0.747 (0.585, 0.909) [1832]{5617}	< 0.01	0.64	0.369 (0.298, 0.440) [1367]{5321}

CI, confidence interval; MRS, marginal rate of substitution (willingness to pay, £). The MRS was computed by dividing each coefficient by the coefficient for cost of attending appointments. The coefficients are rounded and therefore MRS values are not identical to the ratio of the coefficients shown in the Table ^1^ P-value from χ^2^ test that the coefficients across the three groups are the same. ^2^ P-value from χ^2^ test that the coefficients for patients and carers are the same. P-value from χ^2^ test that all coefficients for all three groups are the same is < 0.01; for patients and carers only it is 0.48. ^3^ Standard error of the MRS, calculated using the delta method

### Relative importance of the attributes

Over the range of levels included in the DCE, access to a specialist centre was the attribute valued most highly by patients and carers, followed by clinical expertise, then access to health records, then the cost of attending all appointments over one year, then having documented emergency plan, followed by the role of care coordinator. For health care professionals access to health records was valued most highly, followed by documented emergency plan, then access to a specialist centre, then the cost of attending all appointments over one year, followed by clinical expertise and the role of care coordinator. This analysis of the relative importance of the attributes is preferred to the simple attribute ranking as it accounts for the levels of the attributes.

### Marginal rates of substitution

As an indication of their strength of preference, and the value they put on each attribute, patients and parents/carers were willing to pay £2509 for access to a specialist centre; £2470 for a consultant who was a medical expert in the patient’s condition; £2442 for electronic health records that were immediately accessible to staff; £1367 for a document emergency plan; and £1306 for the support of a care coordinator where the patient/carer decided how they wished to be supported (Table 3). Health care professionals were willing to pay £1864 for electronic health records that were immediately accessible to staff; £1832 for a documented emergency plan; £1802 for patient access to a specialist centre; £1252 for a consultant who was a medical expert in the patient’s condition; and £1131 for a care coordinator who entirely coordinated care on behalf of the patient. These MRS values reflect the relative importance of the attributes, shown above.

### Predicted probabilities

**Fig. 3:**
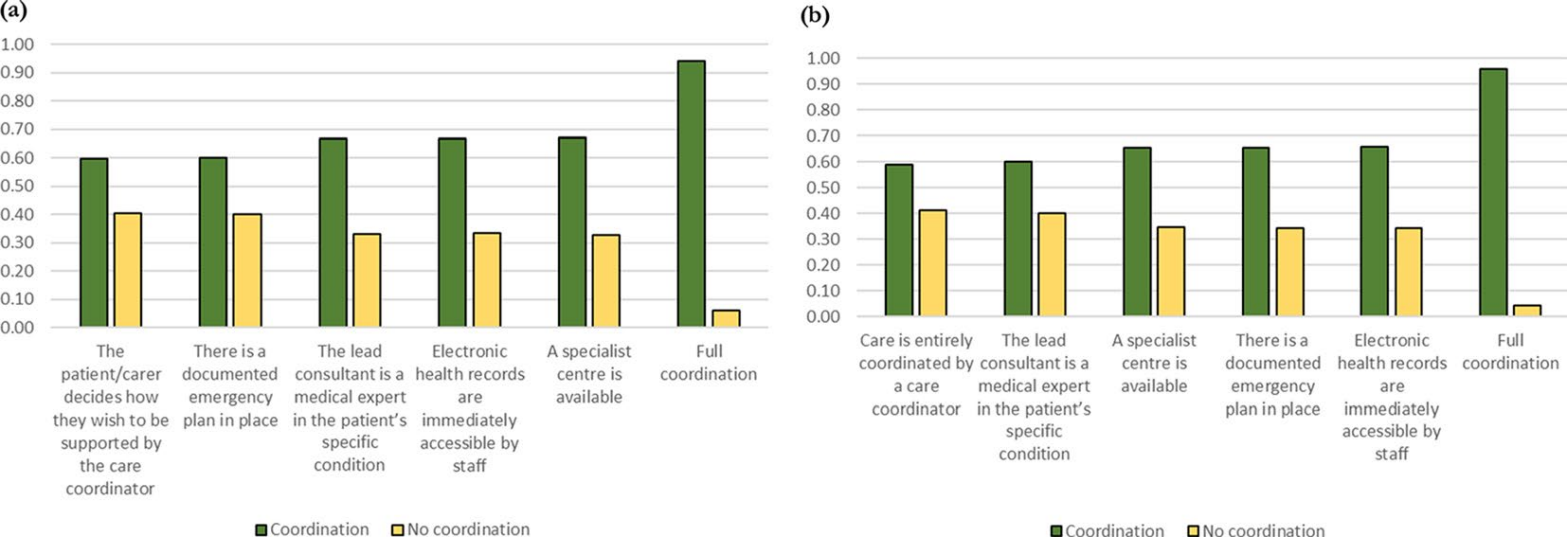
Predicted probabilities of choosing coordinated services. (**a**) Patients and parents/carers combined. (**b**) Health care professionals. No coordination: health records are not shared; the lead consultant is a medical expert in the area of the body primarily affected by the patient’s condition (e.g., neurologist); care is provided without the support of a care coordinator; a specialist centre is not available; there is not a documented emergency plan in place. Full coordination: electronic health records are immediately accessible to staff; the lead consultant is a medical expert in the patient’s specific condition; the patient/carer decides how they wish to be supported by the care coordinator (patients/carers) or care is entirely coordinated by a care coordination (health care professionals); a specialist centre is available; there is a documented emergency plan in place. All other coordination scenarios are as for no coordination except for the attribute indicated. In all scenarios the cost to patients and carers of attending all health care appointments over one year is held constant at £1000. Scenarios are ordered from left to right in ascending order of magnitude of the predicted probability of choosing the coordination service (note the ordering is different for patients and carers combined and health care professionals)

The probability that respondents would choose a service with different types of care coordination versus no coordination is shown in Fig. 3. Compared with a service with no coordination (health records are not shared; the lead consultant is a medical expert in the area of the body primarily affected by the patient’s condition (e.g., neurologist); care is provided without the support of a care coordinator; a specialist centre is not available; there is not a documented emergency plan in place), respondents had a higher probability of choosing a service that had any of the individual attributes of coordination. For patients and parents/carers the probabilities were 0.60–0.67 depending on which individual attribute was selected, with the attributes ranked in terms of their predicted probability in the same order as the relative importance above (Fig. 3(a)). If a service achieved all of the attributes of coordination the probability that patients and carers would prefer to use that service was 0.94. For health care professionals the probabilities for each individual attribute ranged from 0.59 to 0.66, for a service that achieved all of the attributes of coordination the probability was 0.96 (Fig. 3(b)). When coordination reduced costs compared with no coordination the probability that respondents would choose a service with the different types of care coordination increased, and vice versa (Supplementary material).

## Discussion

### Key findings

Patients, parents and carers and health care professionals all preferred services where: the cost of attending appointments was lower; electronic health records were immediately accessible to staff; the lead consultant was a medical expert in the patient’s specific medical condition; care was provided with the support of a care coordinator; a specialist centre was available; and there was a documented emergency plan in place. Preferences were found to be consistent with better coordination of care, though there were some differences between the preferences of patients and parents/carers and health care professionals. The probability that participants would choose a service with all the elements of coordination studied in place was high. All participant groups were prepared to make trade-offs for better care co-ordination. For example, patients and parents/carers were willing to pay £2509 for access to a specialist centre, £2470 for a consultant who was a medical expert in the patient’s condition, £2442 for electronic health records that were immediately accessible to staff, £1367 for a documented emergency plan and £1306 for the support of a care coordinator.

### How the findings relate to previous research

There are several studies that have explored how people affected by rare diseases would like their care to be coordinated, though these tend to focus on single options, such as care coordinators [26] or specialist centres [27]. We are not aware of any studies that have compared between multiple aspects, and none of these have used a DCE-based approach. Previous research using different study designs has highlighted the importance of care coordinators [26], specialist centres, [27, 28] and care plans [29] to support coordination of care for rare conditions.

### Implications

Our findings also have implications for the ways in which care for people with rare diseases might be co-ordinated. Following the publication of The UK Rare Diseases Framework [6], the four devolved nations of the UK are developing action plans that set out how the four priorities identified in the framework will be addressed (note that the third priority is ‘better coordinated care’) [7–10]. Our findings have potentially useful implications for this work, for example by identifying elements of care coordination that matter most and ranking them in order of importance. The trade-offs from the DCE could also be used to value the potential benefits of different models of care coordination. For example, the willingness to pay for each aspect of care coordination is a measure of the value of the benefit of each aspect on average per patient. The willingness to pay could be summed across all patients receiving that aspect of care and balanced against the total costs of providing that aspect of care in a future cost–benefit analysis.

### Limitations

Several limitations are acknowledged. DCEs elicit hypothetical choices, and therefore might lack external validity if individuals do not make the same choices in real-life situations. Some aspects of the DCE might be difficult for respondents to understand, such as the forced choices between services, probabilities and clinical concepts. The data used in this study were collected in 2019 and the delivery of health care is likely to have changed since then; this may also affect have affected coordination of care. For example, the use of remote methods of coordination such as digital information sharing, and virtual clinics and care coordination appointments have been accelerated by the COVID-19 pandemic [30]. The representativeness of the samples used might be limited by the recruitment strategies, yielding potential sampling bias; for example, there was a high proportion of female patients and parents/carers. The modal education category was those who were educated to degree level or higher, and it is unclear if costs would have been the least important attribute if, for example, the sample was on average less well educated. While the overall sample size was large, we obtained fewer responses from parents/carers and health care professionals than targeted. There might be other components of coordinated care that are important but were not included in the present analysis; unfortunately, the number of attributes that can be included in a DCE is limited by the amount of data that participants can process. The nature of our piloting work meant we were unable to produce initial estimates of the model coefficients, which could have been used to inform the final study design – initially, the coefficient parameters were assumed to be zero. Preferences might vary by sub-groups within our study groups (e.g. parents of children affected by rare diseases versus carers of adults with a rare disease), but sample size considerations make sub-group analyses problematic.

### Further research

This study provides new evidence on the elements of care coordination that matter to people affected by rare diseases. Further research would be beneficial to develop different models based on people’s preferences as described in this study, describing how care for people with rare conditions could be coordinated. These models could then be the focus of further formal evaluation. Further research would also be helpful to understand the reasons for the differences in preferences between patients and parents/carers on the one hand and health care professionals on the other.

## Conclusion

The findings of this study highlight that people value better coordinated care, in line with policy documents emphasising commitments to coordinated care for people affected by rare diseases [6]. These findings are relevant to policy-makers, service planners and providers who are designing services for people affected by rare conditions; they show the factors that could be included in service provision as ways of improving the coordination of care.

## Electronic Supplementary Material

Below is the link to the electronic supplementary material.

Supplementary Material 1

## Data Availability

Not applicable.

## Abbreviations

DCE: Discrete Choice Experiment
PPIAG: Patient and Public Involvement Advisory Group
NHS: National Health Service
NIHR: National Institute for Health Research
SWAN: Syndrome Without A Name
