# Supplementary material for: Preferences for coordinated care for rare diseases: discrete choice experiment
Source: Orphanet J Rare Dis. 2024 Sep 9;19:332. doi: 10.1186/s13023-024-03353-0 (PMC11386106; doi:10.1186/s13023-024-03353-0)
Supplement: Supplementary file 1 — Supplementary Material 1 [file 13023_2024_3353_MOESM1_ESM.docx]

**Supplementary material**

**Figure S1. Predicted probabilities of choosing coordinated services: low cost for coordination, high cost for no coordination**

| 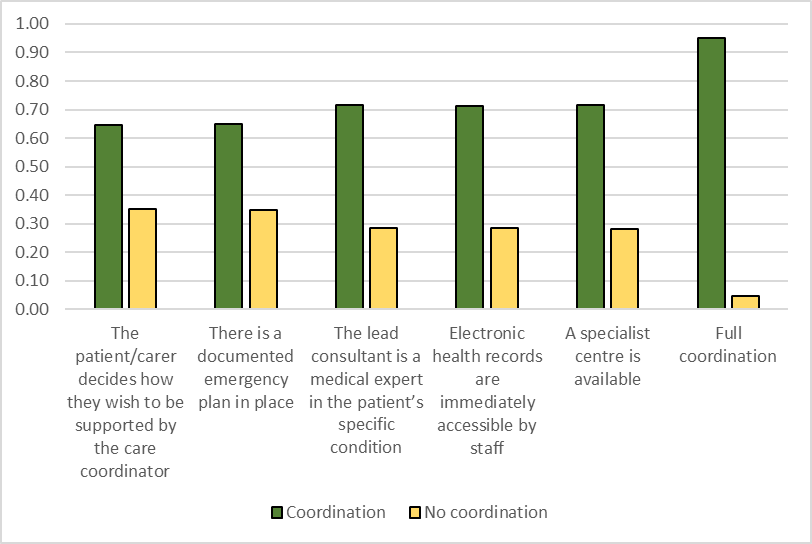 | 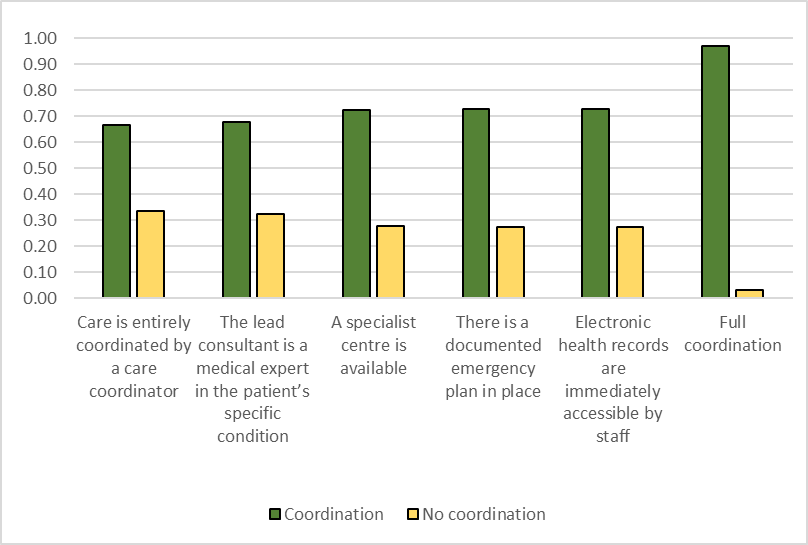 |
| --- | --- |
| **(a) Patients and parents/carers combined** | **(b) Health care professionals** |

No coordination: cost to patients and carers of attending all health care appointments over one year is £2000; health records are not shared; the lead consultant is a medical expert in the area of the body primarily affected by the patient’s condition (e.g., neurologist); care is provided without the support of a care coordinator; a specialist centre is not available; there is not a documented emergency plan in place. Full coordination: cost to patients and carers of attending all health care appointments over one year is £200; electronic health records are immediately accessible to staff; the lead consultant is a medical expert in the patient’s specific condition; the patient/carer decides how they wish to be supported by the care coordinator (patients/carers) or care is entirely coordinated by a care coordination (health care professionals); a specialist centre is available; there is a documented emergency plan in place. All other coordination scenarios are as for no coordination except for the attribute indicated. Scenarios are ordered from left to right in ascending order of magnitude of the predicted probability of choosing the coordination service (note the ordering is different for patients and carers combined and health care professionals).

**Figure S2. Predicted probabilities of choosing coordinated services: high cost for coordination, low cost for no coordination**

| 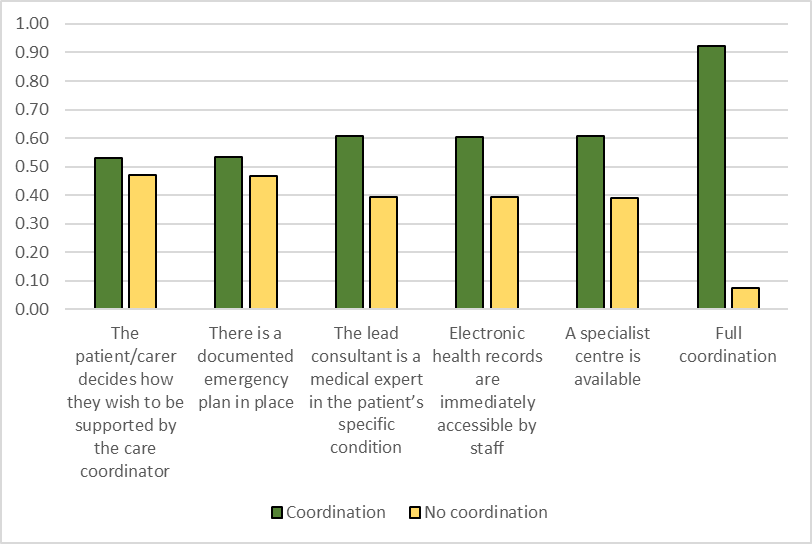 | 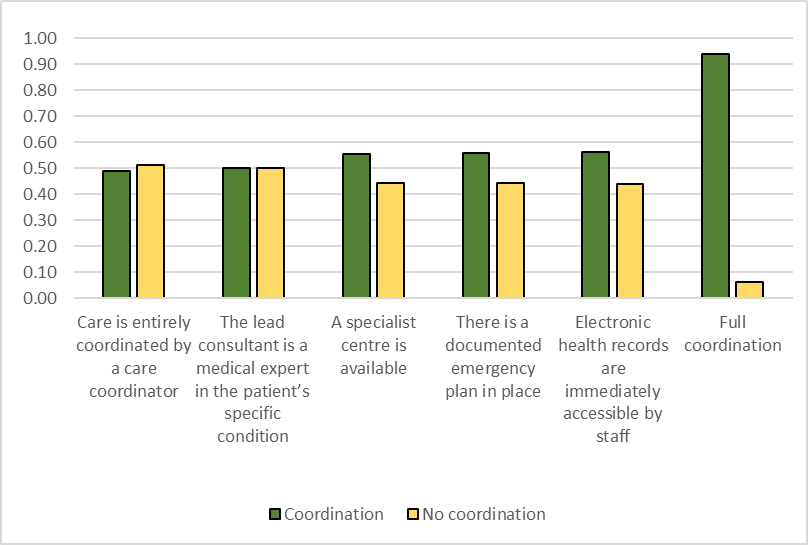 |
| --- | --- |
| **(a) Patients and parents/carers combined** | **(b) Health care professionals** |

No coordination: cost to patients and carers of attending all health care appointments over one year is £200; health records are not shared; the lead consultant is a medical expert in the area of the body primarily affected by the patient’s condition (e.g., neurologist); care is provided without the support of a care coordinator; a specialist centre is not available; there is not a documented emergency plan in place. Full coordination: cost to patients and carers of attending all health care appointments over one year is £2000; electronic health records are immediately accessible to staff; the lead consultant is a medical expert in the patient’s specific condition; the patient/carer decides how they wish to be supported by the care coordinator (patients/carers) or care is entirely coordinated by a care coordination (health care professionals); a specialist centre is available; there is a documented emergency plan in place. All other coordination scenarios are as for no coordination except for the attribute indicated. Scenarios are ordered from left to right in ascending order of magnitude of the predicted probability of choosing the coordination service (note the ordering is different for patients and carers combined and health care professionals).
